# Geographical isolation, mental health, economic stress and firearm suicide in Harris County, Texas

**DOI:** 10.1016/j.cities.2024.105623

**Published:** 2025

**Authors:** Ned Levine, Bindi Naik-Mathuria, Cary Cain, Abiodun Oluyomi, Jeff Carter, Aaron Martinez, Lisa Pompeii

**Affiliations:** a Ned Levine & Associates in Houston, TX, United States of America; b University of Texas Medical Branch in Galveston, TX, United States of America; c Department of Pediatrics in the Division of Public Health Pediatrics at Baylor College of Medicine in Houston, TX, United States of America; d Section of Epidemiology and Population Sciences in the Department of Medicine at Baylor College of Medicine in Houston, TX, United States of America; e University of Houston, Texas Children’s Hospital in Houston, TX, United States of America; f University of Houston – Clear Lake, Texas Children’s Hospital in Houston, TX, United States of America; g Cincinnati Children’s Hospital Medical Center, Division of Patient Services Research, Department of Pediatrics, University of Cincinnati, OH, United States of America

**Keywords:** Suicide, Firearms, Geographic isolation, Mental health, Economic stress

## Abstract

Combining medical and Harris County Medical Examiner records with socio-economic data, 750 firearm suicides that occurred between 2018 and 2020 in Harris County, Texas were studied to understand the interaction of geographical isolation with mental illness, economic stress, and alcohol abuse. Spatial variation in the shooting locations by distance from downtown Houston explored center-periphery differences. Regression modeling by census block groups analyzed the relationship between firearm suicides per capita with neighborhood factors.

Males were more likely to die by firearm suicide as were persons of non-Hispanic White ethnicity. Almost half had a mental health diagnosis or suspicion, in particular depression. The firearm suicide rate increased with age and by distance from downtown Houston as did depression and anxiety disorder. More firearm suicides per capita occurred in block groups farther from downtown Houston and in those that had higher poverty rates and more bars.

The results suggest that firearm suicides are associated with an interaction of aging, geographical isolation, mental illness, and economic stress. There is a need for increased outreach, community services, and mental health services in suburban and rural areas as well as a need to improve firearm safety policies.

## 1. Introduction and aims

Suicide is a leading cause of death (CDC, 2024a). Suicide rates have been increasing in the United States, in the United Kingdom, and in some Eastern Europe and South American countries (Bertuccio et al., 2024; CDC, 2023). Further, the problem has become acute for youth in those countries where youth suicide rates have been rising faster than the national rates (Curtain & Garnett, 2023). These are national comparisons. However, it is important to understand how suicide rates vary within a metropolitan area and in particular neighborhoods since prevention requires specific, targeted interventions.

Geographical separation is a critical factor for isolating individuals and which, in turn, could exacerbate mental health problems that might dispose people to consider suicide. The primary goal was to show how geographical isolation along with mental health and economic stress affects suicide by firearms in Harris County, Texas. We describe individual characteristics of 750 individuals who died by suicide through firearms in Harris County (TX) between 2018 and 2020. Mental health characteristics of individuals that were identified in medical records are discussed. We model firearm suicides spatially to show how particular neighborhoods, especially those that are geographically distant, could exacerbate individual tendencies towards suicide.

A secondary goal was to show how integrating medical and medical examiner records with socio-economic data can lead to an understanding of how the urban environment interacts with personal factors to increase the likelihood of suicide. Unfortunately, there are few studies that have linked these two types of information in explaining suicide.

## 2. Theoretical background

Social isolation has long been known to be a factor in depression and suicide (Calati et al., 2019; Ghadapasha et al., 2024; Motillon-Toudic et al., 2022) and has been discussed since Durkheim’s classic study (1897). Recent studies have shown negative health outcomes for those who are socially isolated. One meta-analysis analyzed forty systematic studies that examined the relationship between social isolation and health outcomes and identified significant associations with increased mortality from all causes, particularly from cardiovascular diseases (Leigh-Hunt et al., 2017). Narrative systematic reviews suggested associations with poorer mental health outcomes as well.

In addition, a meta-analysis of fifty-one individual studies examined the association of social support, social networks, or social connectedness with depression. The strongest and most consistent findings were significant protective effects of perceived emotional support, perceived instrumental support, and large, diverse social networks (Santini, Koyanagi, Tyrovolas, Mason, & Haro, 2015). Little evidence was found on whether social connectedness by itself was related to depression as was also the case for negative interactions.

An earlier meta-analysis of thirty-seven individual studies looked at the relationship between social relations and depression in older adults. Lack of social support, poor quality of relations, and absence of confidants were identified as factors that were significantly associated with depression (Schwarzbach, Luppa, Forstmeier, König, & Riedel-Heller, 2014).

### 2.1. Relationship between Geographical Isolation and Social Isolation

Geographic isolation is conceptually different from social isolation though there is a strong relationship between the two. Of course, it is possible for individuals in densely populated areas to become socially isolated just as it is possible for individuals in more remote areas to become socially active. Nevertheless, generally, there is a positive association between geographical isolation and social isolation.

In the central cities of most American metropolitan areas, population density is much higher with the distance between residential populations and commercial and community amenities being much closer than in suburban or rural areas. Similarly, the suburbs include large tracts of single-family units with little multi-family housing. Commercial areas are usually separated by substantial distances and are not usually within walking distance (Gilderbloom, Riggs, & Meares, 2015; Solomon, 2024; Tomer & George, 2023).

Further, despite substantial decentralization of employment, there are fewer job opportunities and social activities available in the suburbs and rural areas (Couillard & Foote, 2019; Fry, 2020; Shearer et al., 2019; Zuvanich, 2023). Transportation is a critical component with automobile travel being almost necessary to get around in suburban and rural areas since there is limited public transit available (Cervero, 1993; Cox, 2018; Isaacs, 2024; VTO, 2012; Xing, Rodier, Harold, & Sintetos, 2022). Consequently, amenities and social services are typically more geographically separated in suburban and rural areas and are more difficult to access for people needing them.

Rural areas have been shown to have higher suicide rates than urban areas in the United States (Ingraham, 2018; Kegler, Stone, & Holland, 2017; Miller et al., 2012; Branas et al., 2004). Further, these urban-rural differences in rates have increased over time (Harne, 2018; JAMA, 2017; Rossen, Hedegaard, Khan, & Warner, 2018; Branas et al., 2004). One study found that the rate of social isolation in non-urban areas was 21 % higher compared to urban areas after adjusting for covariates, though the relationship was not statistically significant (Lynch et al., 2021).

Variation in rates of mental illness (also known as mental disorder, mental health condition, or psychiatric illness; WHO, 2022) was examined in 2,960 U.S. counties with Census and CDC data as a function of geographic and socio-economic variables. Most variation in county mental illness rates were accounted for by the independent effects of geographic isolation, socioeconomic status, income inequality, and other variables (Hudson & Doogan, 2019).

One study reported higher risk of suicide among older persons in Victoria, Australia, particularly socially and geographically isolated older men (Pate, 2014). Another study reported that individuals who lived in remote areas or who were geographically separated from family and friends due to job duties, such as military service, experienced feelings of isolation (Tulane, 2020). Even within a metropolitan area, suburban areas have been shown to have higher suicide rates than those in the central city. A study of Harris County, Texas showed a gradient of suicide risk with distance from downtown Houston (Levine et al., 2020).

Stevens, Carson, and Wall (2024) summarized how geographical isolation can exacerbate practical and mental health problems of individuals. These include financial, transportation, technological, cognitive, and sometimes cultural barriers to active participation in nearby communities. Further, geographical isolation can limit access to health, legal and dispute resolution services, as well as limit information about social and community resources.

### 2.2. Distance from the city center as a measure of geographical isolation

While there is not a single measure that can define geographical isolation, distance from the metropolitan center captures much of the effect of geographical separation and can be an index of how separated households and individuals are from others, all other things being equal. Usually, population densities decline with distance from a metropolitan center as do other characteristics that correlate with population density, primarily as a function of the distribution of activities based on land rent (Alonso, 1964; Egan & Nield, 2000; Filatova et al., 2009).

Further, even controlling for population density, the risk of several types of incidents have been shown to vary by distance from the central city including motor vehicle crash rates (Levine, 2011) and violent crime rates (Kneebone & Raphael, 2011). One would expect that suicide rates will vary by distance from the central city as well as mental health rates.

### 2.3. Other risk factors

Other associations with suicide that have been well documented include being male (CDC, 2023; Kegler et al., 2017), being older (especially 85 or over; CDC, 2023; SPRC, 2019), and being unmarried, divorced or separated (Lehman, 2020; Akbarizadeh et al., 2019).

In the United States, there are clear disparities by ethnicity with Native Americans having the highest overall suicide rate followed by non-Hispanic Whites and non-Hispanic Native Hawaiians/Pacific Islanders (CDC, 2023; Naik-Mathuria, Cain, Elizabeth, Chen, & Pompeii, 2023). Veterans have a high suicide rate, varying from 1.5 to 2.5 times that of the general population (CDC, 2022; VA, 2022).

A history of mental health problems, especially depression, is a strong risk factor associated with suicide for both children and adults (Brådvik, 2018; Cuesta, Montesó-Curto, Metzler Sawin, et al., 2021; McLoughlin et al., 2019). Depression leading to suicide has also been linked to adverse childhood experiences, poor social support, and isolation (Gloger et al., 2021; Merrick et al., 2019). On the other hand, the results for alcoholism are uneven with some studies supporting a relationship with suicide (Inskip et al., 1998) and others not (Heu et al., 2018).

Economic insecurity has been associated with increases in suicides and substance-abuse deaths especially for older, non-Hispanic White persons without college degrees (Case & Deaton, 2017, 2023). Finally, access to firearms significantly increases the risk of suicide (Mann & Michel, 2016; Studdert et al., 2020). Without that access, suicide would be much more difficult to achieve. We will come back to this point in the discussion.

In short, examining the interaction between social and personal factors is important for understanding the etiology of suicide in the U.S. and other countries because neither mental illness nor social isolation nor economic insecurity by themselves will trigger suicides. Further, the easy availability of firearms in the U.S. makes it easy for individuals to seek suicide. Unlike illegal drug use, alcohol abuse or other self-destructive behaviors, there is little chance of withdrawing from such behavior. Therefore, preventive actions must precede suicide decisions to be effective.

### 2.4. Hypotheses

There is an interaction between geographical isolation and several individual factors. Specifically, it is hypothesized that:

1. H0: There is a positive association between firearm suicide risk (relative to population) and distance to the central city.
  H1: There is no relationship between firearm suicide risk and distance to the central city.
2. H0: There is a positive association between population rates of mental illness with distance to the central city.
  H1: There is no relationship between population rates of mental illness with distance to the central city.
3. H0: Economic stress can exacerbate the effect of geographic isolation and mental illness on suicide.
  H1: There is no relationship between economic stress and geographic isolation or mental illness.
4. H0: In turn, increasing population rates of mental illness can increase social isolation and, thereby, increase suicide rates even more.
  H1: There is no relationship between increasing rates of mental illness with social isolation.

This latter hypothesis cannot be tested without time series data. But it follows from the above relationships.

## 3. Methods and materials

### 3.1. Geography

Harris County, Texas is the third largest county in the U.S. by population (4.7 million in 2021) and is within a metropolitan area of more than 7.5 million (Houston Chronicle, 2024). It includes the City of Houston which is the fourth largest city in the U.S. (2.3 million in 2021). It is a primarily urban county but has a large suburban population which is growing very rapidly (Zuvanich, 2023). The U.S. Census Bureau does not define rural areas but consider them as areas that are not urban, defined as areas with at least two thousand housing units or having a population of at least five thousand (U.S. Census Bureau, 2022). Also, they no longer distinguish urban clusters (i.e., the core of the urban area) from the urbanized area but consider all areas within as being urban. Thus, suburban is not defined either. Nevertheless, there are areas within Harris County that are rural. It has been estimated that as much as 27 % of the county is rural (Greater Houston Partnership, 2024).

### 3.2. Database

Data on individuals who died by firearm suicide between January 1, 2018 and December 31, 2020 were collected from the Harris County Medical Examiner’s Office, housed within the Harris County Institute of Forensic Sciences. In turn, the medical examiner data incorporated information from hospital medical records, where available. Death records reviewed included forensic investigator notes of circumstances at the scene of the death along with interviews with family members, medical history (if available), and police reports. Each case was reviewed for shooting details. Data were collected on patient demographics, health insurance status, the address of residence and shooting locations, prior and existing mental health conditions and comorbidities, alcohol and substance abuse, stressors and circumstances leading to the event, and access to firearms.

For mental health status, five separate conditions were identified: depression, anxiety disorder, bipolar disorder, post-traumatic stress disorder (PTSD), and schizophrenia. All data were abstracted into a secure REDCap^®^ database (REDCAP, 2023). All procedures were approved through the Institutional Review Board (IRB) at Baylor College of Medicine. Residence and shooting locations (if different) were geocoded to allow spatial analysis.

### 3.3. Data analysis

Three types of statistical analysis were conducted. First, there was a descriptive analysis of the cases and a comparison with national and Harris County rates, where possible. A major problem was the substantial amount of missing information from the data set, due to the investigator’s or hospital staff’s inability to verify decedent characteristics or history. Therefore, the demographic characteristics are presented as proportions with uncertainty, calculating them relative to both the total sample and only those cases that were known. These correspond to two competing assumptions about the missing data, either that the missing information indicated the characteristic was not operative or that the missing information follows the same distribution as that which is known. This is different from sampling error which is always present. Statistical comparisons with reference criteria were made to the most conservative of that uncertainty range (i.e., the value in the uncertainty range that was closest to the reference criterion value).

Second, there was spatial analysis of the suicide locations to identify center-periphery differences in firearm suicides. The number of firearm suicides would be expected to decline with distance from the central city (downtown Houston) primarily because population densities decline with distance. But, relative to population, changes in firearm suicide risk with distance were examined.

Third, a multivariate spatial regression model was constructed of firearm suicide locations by census block groups to identify social factors that could be indicators of suicidal behavior. In Harris County, 2020 census block groups average about thirty-five actual blocks and are the smallest geographical unit for which socio-economic data are available. In the central city, lot sizes are usually smaller as are city blocks. Also, central neighborhoods are denser with more multi-unit residential buildings than in the suburbs. Of the 750 firearm suicides, 739 were able to be geocoded and assigned to block groups that they fell within.

Next, the number of firearm suicides per block group was related to a set of plausible covariates (shown in Table 1). There are demographic, economic, educational, land use, and community cohesion variables. There is also a geographical separation variable of distance from downtown Houston (in miles). This variable measures broad spatial differences due to living centrally versus more peripherally in the county.

### 3.4. Statistical model

Because the distribution of firearm suicides is extremely skewed with 83 % of the block groups having no suicide incidents, the number of firearm suicide cases relative to population for each block group was related to the independent variables using a Poisson-Gamma (negative binomial) statistical model with population being fixed as a rate (Besag, Green, Higdon, & Mengersen, 1995). This is called an *exposure* model (or, sometimes, an *offset* model). Because the dependent variable is a rate (firearm suicides per capita), it cannot be entered directly into a Poisson-based model, which is a count model. Instead, the exposure variable (population) must be treated as an offset. Appendix A provides details of the model.

Two runs were made. In the first, the number of firearm suicides per capita was tested against all the independent variables in Table 1. This is called the *full model*. In the second, the number of independent variables was reduced so that the remaining variables were all statistically significant and relatively independent. This is called the *reduced* (or *parsimonious*) model.

To minimize multicollinearity in identifying independent variables for the reduced model, the tolerance criteria was used (Goldberger, 1991). The higher the tolerance of a predictor variable, the greater is its statistical independence. For the reduced model, any independent variable with a tolerance less than 0.7 was dropped.

## 4. Results

In the three-year period, 750 cases of firearm suicide occurred. Fig. 1 shows a map of the shooting locations of 739 firearm suicides that were geocoded.

Handguns were the most common type of firearm used among this sample (80 %) followed by shotguns (6 %), rifles (4 %) and unknown firearms (10 %). The location of guns prior to the shooting was unknown in 85 % of the cases with, for those details known, the locations including drawers, closets, cars, safes, and on top of tables or dressers. Whether the gun was locked or secured was also not consistently documented in the records.

### 4.1. Demographic characteristics

Most firearm suicides were by males (86 %). The firearm suicides increased with age, though peaking at ages 55–64 (*r* = 0.82; *p* ≤ .001). Fig. 2 shows the relationship.

More than half (59 %) were persons who were non-Hispanic White while 20 % were Hispanic, 14 % were Black/African American, 6 % were Asians/Asian-Americans and 1 % were ‘Other’ (including one Native American). The distribution of firearm suicides differed from the 2020 Harris County ethnic distribution: 27 % non-Hispanic White, 45 % Hispanic, 21 % Black/African-American, 8 % Asian and 1 % American Indian/Alaskan Native. Non-Hispanic Whites were 2.2 times more likely to die from firearm-related suicide compared to their share in the general Harris County population (approximate 95 % C.I.: 2.06–2.33), while Hispanics (RR: 0.4; 95 % CI: 0.65–0.87), Black/African-Americans (RR: 0.7; 95 % CI: 0.42–0.61), and Asians (RR: 0.8; 95 % CI: 0.17–0.30) were at a reduced risk.

Marital status information was obtained for 79 % of the cases. Married persons were the most common marital group who died from suicide by firearm, but there was an uncertainty range of 39.6 %–50.0 %. Single persons were the next largest marital group with an uncertainty range of 31 %–39 % followed by divorced and separated individuals (7 %–9 %) and widowed individuals (1.7 %–2.1 %). The Harris County 2023 distribution of marital status by ages 15 and older was 49.0 % married, single 37.0 %, divorced 9.6 %, and widowed 4.4 % (Houston State of Health, 2023). Comparing the uncertainty range with the county percentages, only the widowed were significantly different (smaller) than the county proportions (Z = −3.07; *p* ≤ .01; Kanji, 2006 p. 26). It is possible that married persons had a lower rate but there were too many missing records to draw a firm conclusion.

There were twenty-one known veterans among the sample. But the military status was known for only 21 % of the individuals. Thus, uncertainty could vary from 2.8 % to 13.2 % compared to 3.2 % of the Harris County population being veterans (U.S. Census Bureau, 2023). No conclusion can be drawn about the suicide rate for veterans in Harris County because of missing data for this variable.

### 4.2. Personal risk characteristics

A mental health diagnosis or suspicion occurred in 43 % of the cases with the most common being depression followed by anxiety disorder, bipolar disorder, PTSD, and schizophrenia. However, missing information created substantial uncertainty.

Medical records indicated that prior alcohol abuse was identified in 53 % of cases while 29 % had prior illegal drug abuse. However, at the time of death, alcohol and drugs were not tested for the majority (99 %) of cases, making any determination of alcohol or drug use in these suicide cases uncertain.

### 4.3. Personal stressors

Recent personal stressors were identified in 81 % of cases, mostly derived from the Medical Examiner’s forensic investigator records. These problems included intimate partner (uncertainty range 29 %–36 %), medical (23 %–28 %), financial (7 %–9 %), job loss (7 %–8 %), death of a loved one (6 %–8 %), and bullying (0.3 %–0.4 %).

Correlations with age were found for several stressors. Those under 18 (usually called ‘pediatric’) and those 65 or older (often called ‘geriatric’) are commonly used age categories. The data determined how those in between were categorized.

Younger persons were more likely to have stress from intimate partners (correlation with age, *r* = −0.21; *p* ≤ .001) while older persons were more likely to have medical problems (correlation with age, *r* = 0.45; p ≤ .001). Those aged 30–49 had the highest percentage of intimate partner stress (41 %; χ^2^ = 8.33, *p* ≤ .0.01) as well as the highest percentage for financial problems (12 %; χ^2^ = 107.26, *p* ≤ .0.001). Medical problems were strongest in the 65 or older age group (55 %; χ^2^ = 117.03, p ≤ .0.001). Those under age 18 had similar low percentages to those 18–29 for all types of stressors.

### 4.4. Center-periphery gradient

The majority of suicides (71 %) occurred at the patient’s home while 11 % occurred in an automobile, often near the patient’s home. For spatial characteristics, there was a center-periphery gradient in the firearm suicide rate (per 100,000 population) with rates higher further away from downtown, in the suburbs and rural areas of the County (Fig. 3). The correlation of the firearm suicide rate with distance from the central city (in miles) was highly significant (*r* = 0.60; *p* ≤ .001). Fig. 4 shows a kernel density map of firearm suicides relative to population in Harris County with the rates quite high in the suburbs.

Further, the rate of diagnosis of mental health conditions for those dying from firearm suicide relative to population increased with distance from the downtown (Fig. 5). The correlation of one or more mental health diagnosis per 100,000 population with unit miles from downtown Houston was highly significant (*r* = 0.64; p ≤ .001). This was particularly true for depression and anxiety disorder where the correlation of those rates (per 100,000 population) with distance was 0.60 and 0.51, respectively.

For example, for those dying from firearm suicide within 10 miles of downtown Houston, 40 % were diagnosed with one or more mental health problems. For those dying by firearm suicide between 10 and 19 miles from downtown Houston, 43 % were diagnosed with a mental health problem. Finally, for those who died by firearm suicide at 20 or more miles from downtown Houston, 45 % were diagnosed with a mental health problem. These differences, while small, were statistically significant (see correlation above).

### 4.5. Neighborhood risk factors

Table 2 presents the output of the two regression models at the census block group level. In the full regression model, the intercept and five significant independent variables were associated with more firearm suicides per capita in block groups: negative coefficients for number of Black persons dying by firearm suicide and number of Hispanic persons dying by firearm suicide while positive coefficients for number of bars, the percent of the electorate who voted in 2016, and the distance of the block group from downtown Houston. In other words, block groups with fewer Black persons, fewer Hispanic persons, more bars, having a higher percentage who voted in 2016, and which were farther away from downtown Houston were more likely to have firearm suicides. However, the negative coefficients do not indicate the existence of a condition that could facilitate firearm suicides, but the lack of those conditions.

In the reduced (parsimonious) model, there were three independent variables that were significant in predicting the number of firearm suicides per capita: 1) A higher percentage of the population living in poverty; 2) More bars; and 3) Greater distance from downtown Houston. All coefficients were positive and statistically significant with low multicollinearity.

## 5. Discussion and conclusion

The aim of this study was to examine the interaction of geographical isolation, mental health and economic stress in exacerbating suicide by firearm. To this aim, we examined 750 firearm suicides that occurred between 2018 and 2020 in Harris County, Texas.

### 5.1. Summary

First, consistent with national data, a large majority of the firearm suicides in the sample were by males (CDC, 2023). Second, the firearm suicide rate (per 100,000 persons) increased with age. Unlike national data that shows ages 85 and older having the highest rates (CDC, 2023; SPRC, 2019), the sample’s peak was for the 55–64 age group. More years of data are needed to see whether these results are consistent.

Third, persons of non-Hispanic White ethnicity showed the highest rate in the sample, consistent with national data (CDC, 2023). Nationally, Native Americans have the highest rate among ethnic groups. But the Native American population is very small in the Houston region, and there were not sufficient numbers of cases to calculate a rate.

Fourth, consistent with other studies (e.g., Brådvik, 2018), mental health conditions were associated with 43 % having been diagnosed or suspected of such conditions, in particular depression. However, missing information created uncertainty about the actual incidence.

Fifth, the firearm suicide rate increased with distance from downtown Houston with higher rates in the suburban and rural parts of the county. This was true at the individual as well block group level. Nationally, suicide rates in rural areas are much higher than in urban areas (Ingraham, 2018; Kegler et al., 2017), particularly for the elderly (e.g., Calati et al., 2019; Motillon-Toudic et al., 2022). As mentioned earlier, geographical isolation is correlated with social isolation, though the two are not identical concepts. Whether this indicates that those individuals in our study who lived farther from the city center were socially isolated or not cannot be determined from the data. Nevertheless, the relationship is consistent with that assumption.

Further, the increase with distance from downtown Houston in mental health conditions relative to population associated with individuals who died by firearm suggests an interaction between geographic isolation and personal mental health problems. For example, persons who are depressed and also geographically isolated may be more prone to suicide than depressed persons who live in neighborhoods with social activities that are closer. While there are alternative explanations for the association, they seem less plausible. It is doubtful whether mental illness is more prevalent in the suburbs than in the central city. Similarly, it is doubtful whether individuals who live in the suburbs are more likely to be diagnosed with depression or anxiety disorders than those who live towards the center (Diagnostic Bias), especially since the bulk of mental health facilities and specialist offices are centrally located in Houston.

In his classic study, Durkheim (1897) showed that urban areas had a higher suicide rate than rural areas. However, the land use patterns of 21st Century suburban and rural communities in the United States are different than those of 19th Century French villages and rural towns. Large tracts with separation between subdivisions is the norm in Houston and many U.S. cities, especially those that developed around the automobile during the latter half of the 20th Century. This separation discourages neighborhood activities and requires most trips to occur by automobile, rather than by walking or bus. This, in turn, can exacerbate social isolation and depression for those who have such a condition. Clearly, more research on the effects of spatial distribution on mental health is needed.

Sixth, the neighborhood analysis showed a relationship between the number of firearm suicides in block groups with distance from downtown Houston as well as higher poverty rates and more bars. Financial stress is important for those under age 50 but less relevant for those older. This suggests that economic stress could be a triggering factor in suicide risk, particularly for those still in their working years. Case and Deaton (2023; 2017) have shown that the mortality rate for older White persons without college degrees has increased over the last two decades, primarily due to increased suicides, drug overdoses, and alcohol-related liver diseases, and has accelerated since the onset of COVID in 2020. The association of a higher percentage of the population living in poverty with the firearm suicide rate in the blocks is consistent with that interpretation.

The biggest increase in suburbanization in the U.S. occurred after World War II with the expansion of automobile ownership and the building of the U.S. highway system (Ames & McClelland, 2002; Nicolaides & Wiese, 2017). For a long time, those who moved to the suburbs were middle class and moderately affluent. However, in more recent decades, suburban communities have experienced an increase in poverty so that roughly 10 % of the suburban population in the U.S. now live in poverty (Kneebone & Berube, 2023; Reiman, 2023; Rockefeller Foundation, 2013).

More neighborhoods in poverty are located centrally in Houston with the highest levels associated with racial and ethnic minority groups. Yet, suicide is rarer in those communities. This supports the hypothesis that economic stress becomes a trigger for suicide, again, when the individual is socially isolated.

The relationship of the number of bars with the firearm suicide rate could suggest that alcohol abuse is an associated factor in the suicides. Around half of these individuals had a prior history of alcohol abuse and about one-fourth had a prior history of drug abuse. Unfortunately, very few were tested for alcohol or drugs after they were deceased.

### 5.2. Policy implications

While these associations are only suggestive and cannot be demonstrated with our data to be determinants, they may indicate that these conditions contribute to suicide. One must keep in mind that suicide is a rare phenomenon and most individuals with mental illnesses or who live in suburban areas do not die by suicide. The variables are predictive for aggregate relationships but not necessarily for individuals. Clinically, they should be seen as potential warning signs, rather than clear diagnostic indicators of a suicide attempt.

#### 5.2.1. Improved suburban and rural outreach

Still, there is a need to reduce social isolation in the suburban and rural areas to reduce the number of suicides. First, improved outreach for individuals who have already been identified as having potential for suicide is necessary. This, of course, requires a hospital or other medical provider to have case workers who can periodically follow up on patients who are suspected of being socially isolated. But, given the aging population and the increased financial resources that are being focused on the elderly, it should be feasible to have more contacts with existing patients.

#### 5.2.2. Encourage more community events in suburban and rural areas

Second, efforts could be made to encourage suburban and rural jurisdictions to facilitate more community events and resources to engage individuals who might be socially isolated. Regional planning agencies are a good vehicle for doing this since they are in contact with most jurisdictions in the region. Efforts are already being made along those lines, at least in the Houston metropolitan area (H-GAC, 2024). However, it would need additional funding from the Federal government or state governments to expand those services. As the population continues to age, such programs will be increasingly needed.

#### 5.2.3. Improve access to mental health Services in Suburban and Rural Areas

Third, given the apparent association of mental illness with suicides (nearly one half), improving access to mental and behavioral health facilities becomes important. Putting mental care facilities closer to where patients are located is critical, especially in the suburbs and in the rural areas. For example, Houston has the largest medical center in the world with more than forty major hospitals located in a central district (Texas Medical Center; https://www.tmc.edu/). Further, many hospitals have created satellite facilities of which there are more than two hundred for a metropolitan population of more than seven million. But most deal with general medicine rather than specialist services. Most mental health facilities and specialist offices are centrally located, with few in the suburbs. There is a lag between the suburban population growth and the availability of these services, leaving gaps in access to mental health services.

It is not easy to access people who may be socially isolated and who, because they are depressed or have another mental illness, may actively try to stay that way. One meta-analysis reviewed sixteen studies that examined the effects of interventions on reducing social isolation (Veazie, Gilbert, Winchell, Paynter, & Guise, 2019). Of the interventions, physical activity interventions showed the most promise at improving the health of older adults; however, the effects were inconsistent and often short term. Another meta-analysis reviewed twenty-seven studies that examined the effectiveness of suicide prevention programs for youth and concluded that follow-up monitoring and the identification and treatment of depression were the leading factors in reducing the likelihood (Fakhari, Azizi, Farahbakhsh, & Esmaeli, 2022). Such efforts are clearly expensive.

In general, financial resources for outreach and other social services are very inadequate in the U.S. so that it becomes difficult to obtain funds or hire a trained workforce who can do those jobs. But local government and human service organizations (including medical facilities) must try to conduct that outreach to improve the lives and reduce preventable deaths as much as possible.

#### 5.2.4. Lethal means restrictions

Suicide by firearm is the leading cause of firearm death in adults in the United States, and the second leading cause in children (Johns Hopkins, 2023). The incidence has increased steadily since 2006, reaching an all-time high in 2022, when almost 27,000 Americans died by firearm suicide (CDC, 2024b). Military personnel and veterans are at higher risk of suicide (DeAngelis, 2022; Pruitt et al., 2019). A recent survey estimated that 36 % of the elderly own a firearm (Gallup, 2020).

In the United States, the U.S. Constitution allows individuals to own firearms for military and self-protective purposes subject to regulation. The Courts have interpreted this as allowing individuals to own guns with few restrictions (mostly regarding military-type weapons and those convicted of felonies for a period after their incarceration). Consequently, the number of guns in the U.S. produced for individual consumption exceeds the total number of persons, excluding weapons for law enforcement and military purposes (Mascia & Brownlee, 2023). Political attitudes have become very hardened with gun advocates unwilling to concede legislative actions that might reduce the number of weapons available.

Moreover, there is no single solution to firearm violence in the United States (see Smart et al., 2024; Webster & Wintemute, 2015; NIJ, 2014). Reducing the number of shootings would involve establishing gun registries and gun user licensing, imposing restrictions on informal gun sales, strengthening child access laws, improving Extreme Risk Protection (Red Flag) laws which temporarily remove guns from people believed to be a danger to themselves or others, better regulating concealed-carry laws, eliminating stand-your-ground laws, restricting assault rifles and high-capacity magazines, increasing after-school programs for high-risk youth, improving security protection at schools and religious organizations, reducing the risk in environments that attract firearm violence (e.g., late night bars), and improving mental health services. Action at various levels of government and in the private sector are all needed. Unfortunately, currently there is little political willingness to address such measures in totality. Legislative bodies tend to take a limited, piecemeal approach.

At an individual level, anticipatory guidance by physicians, who often have frequent contact with persons whom they suspect of showing suicide ideation, should center around safe storage of firearms (storing firearms unloaded and locked with ammunition stored separately) or choosing alternate options for protection rather than a firearm (e.g., dog, house alarm). Additionally, other means for suicide prevention, such as opioid restrictions, are important. Instituting lethal means counseling policies in health and behavioral health care settings should be promoted, especially for people at risk for suicide such as older adults with social isolation or depression (Kivisto, 2022).

A shift in discussing the topic has been to emphasize the concept of ‘safety’ rather than ‘restrictions’ in limiting access to firearms for potential suicides (Stanley, Hom, Rogers, Antestis, & Joiner, 2017). This is a more positive approach that might lessen the objections of gun advocates and allow legislative actions to occur. Even the gun industry promotes firearm safety. Still, just changing terminology will not eliminate the reluctance of gun advocates and lobbyists (e.g., weapon manufacturers) to reduce firearm access. It may take changes in public attitudes that show themselves in elections before substantial changes can occur.

### 5.3. Limitations

There are six limitations to the study. First, the study represents only one geographical area. The conclusions may not be generalizable to other regions of the country. Second, the results may not be generalizable to other countries than the U.S. The suicide rate has decreased in western and northern Europe (though not in the United Kingdom and much of eastern and southern Europe, South America). Countries that limit suburbanization through urban limit lines, greenspaces or other regulatory mechanisms may not show the same increase in the suicide rate with distance from the central city.

Third, as mentioned, there was a large amount of missing information in the medical and forensic records about veteran status, alcohol use, and drug use and substantial missing information about marital status, mental health conditions, and personal stressors. The missing information creates uncertainty about the conclusions. While this is a problem that is common in hospitals throughout the country, the lack of consistent data collection creates substantial uncertainty about statistical relationships. Improved medical and forensic records data collection for the medical records would certainly improve research on this topic.

Fourth, social isolation has not been measured directly, but inferred from the center-periphery gradient of the firearm suicide rates and the mental illness rates. Further tests using more direct measures are needed.

Fifth, not all persons who die by firearm suicides have a mental illness, slightly less than half do. Whether social isolation contributes to suicide for those who do not have mental health problems cannot be easily determined from the data.

Sixth, for the block group analysis there was uncertainty in generalizing from aggregate relationships to individual characteristics. This was especially true for the economic variable that was identified in the neighborhood analysis, namely a higher percentage of the population living in poverty. Unfortunately, the economic situation of these 750 individuals was not known.

## Figures and Tables

**Fig. 1.**
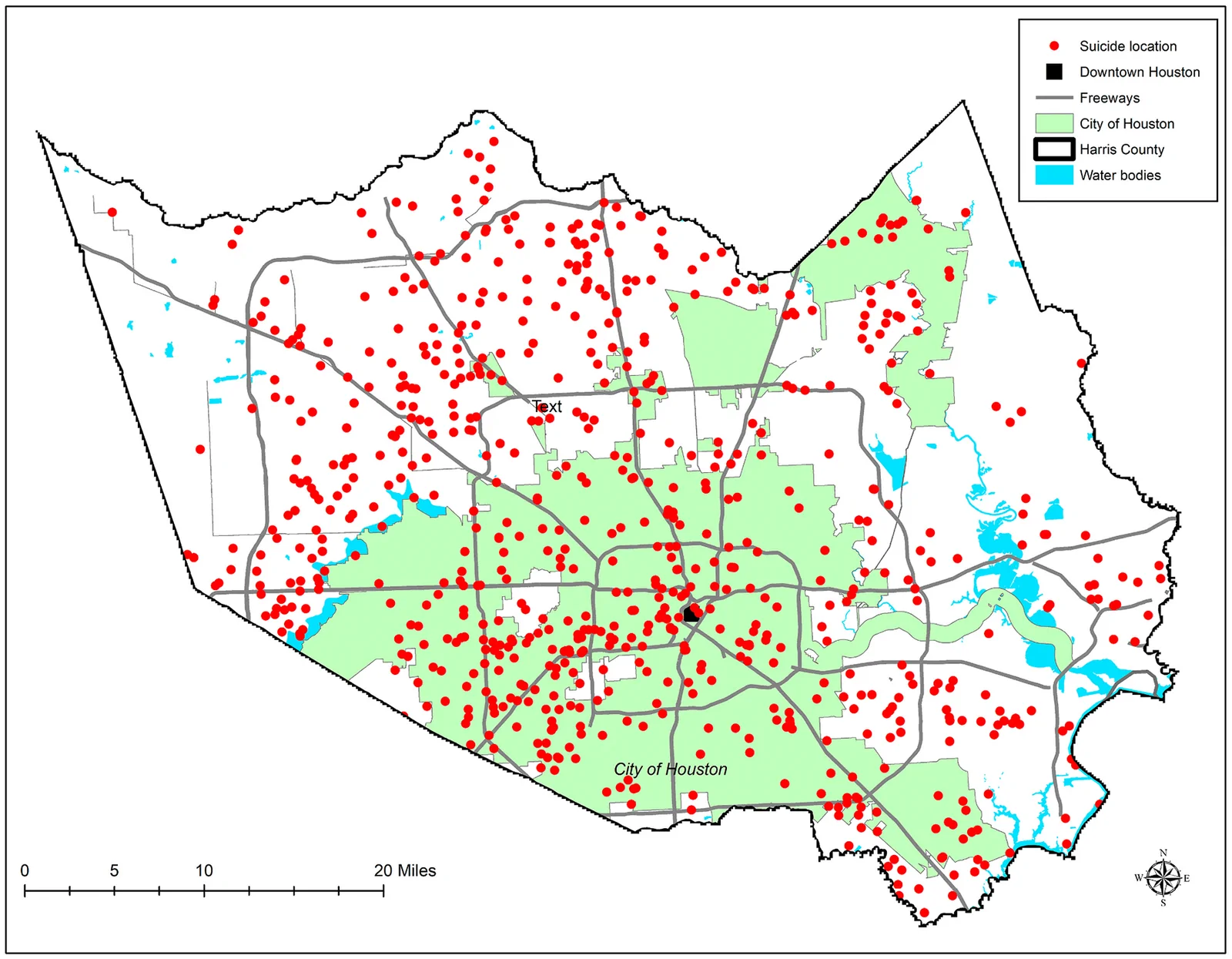
Suicides by Firearm in Harris County, TX: 2018–2020 / N=739 Geocoded Suicide Locations

**Fig. 2.**
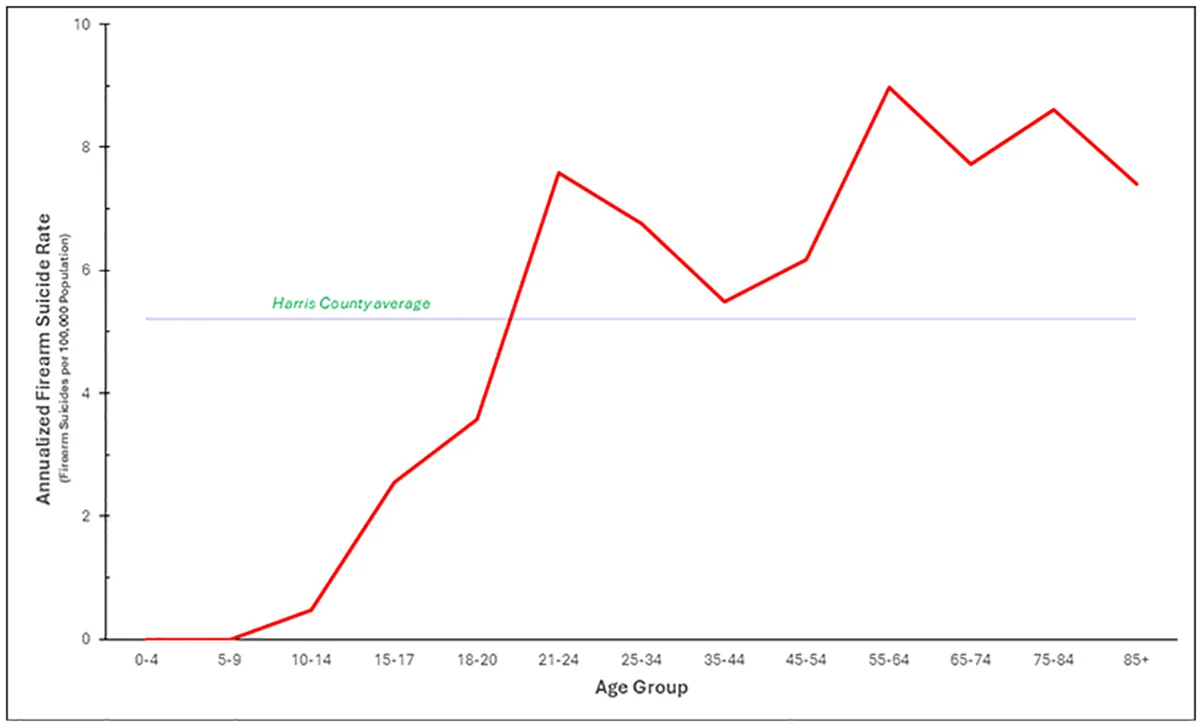
Firearm Suicide Rate (per 100,000 persons) and Age: 2018–2020

**Fig. 3.**
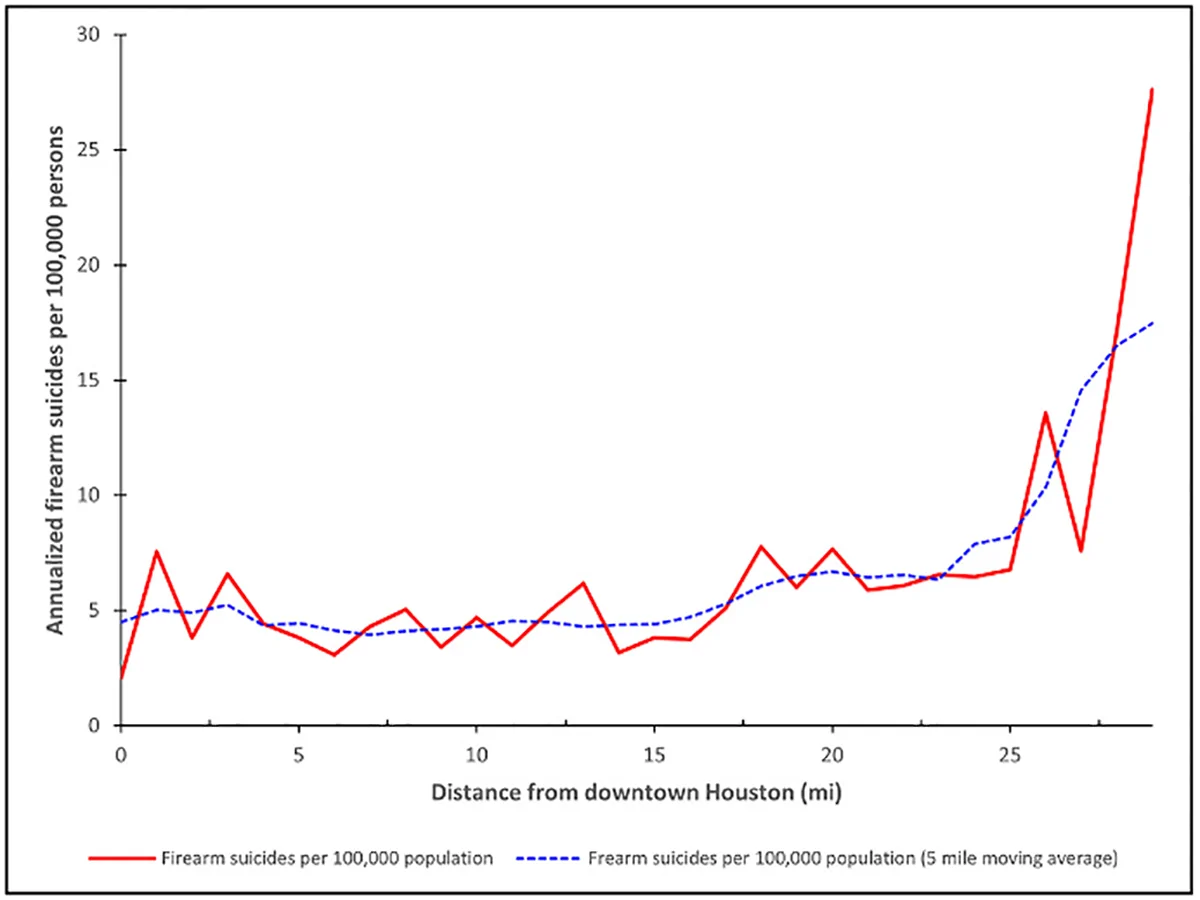
Firearm Suicide Rate (per 100,000 persons) by Distance from Downtown Houston

**Fig. 4.**
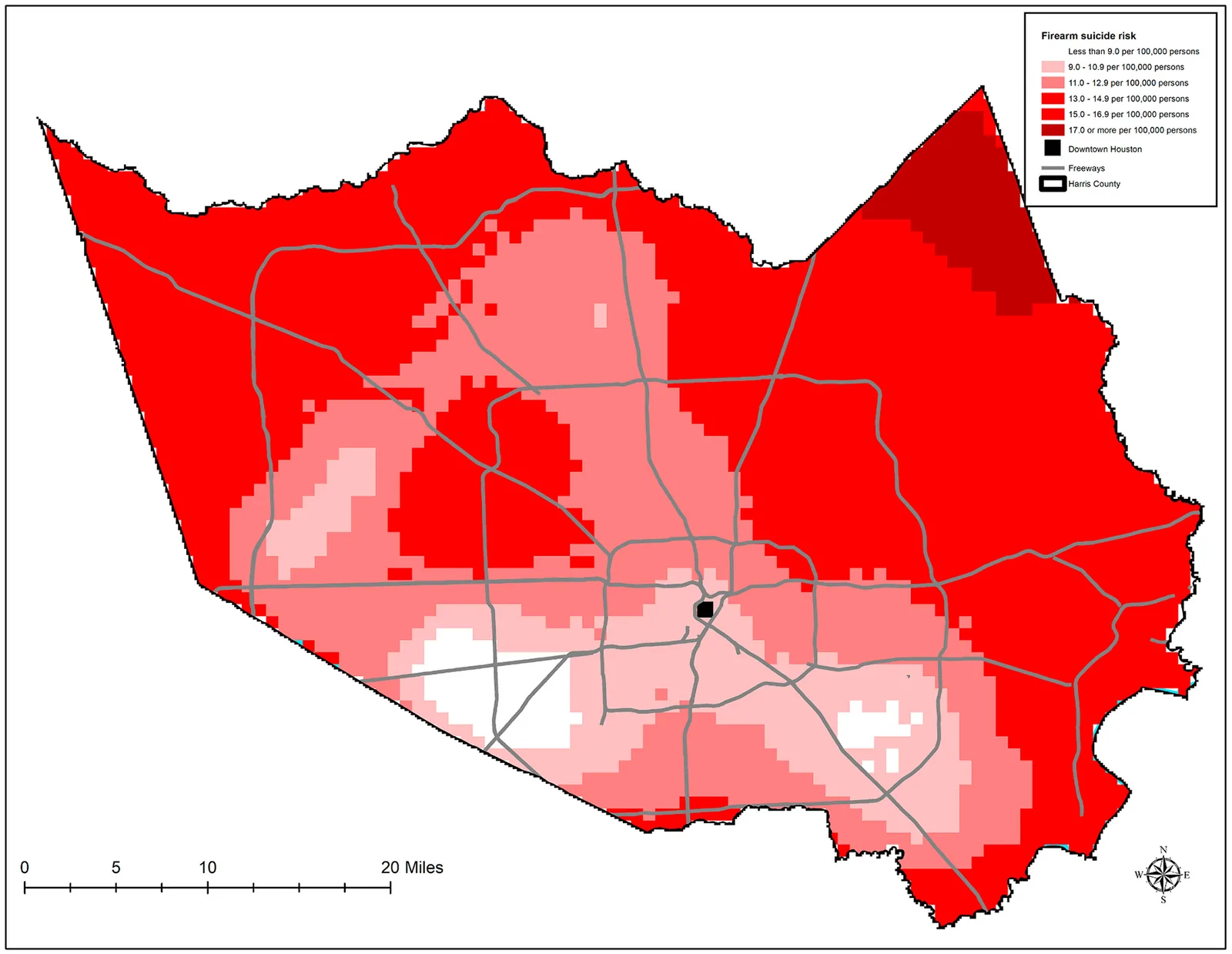
Harris County Firearm Suicide Risk: 2018–2020 / Firearm Suicides Relative to 2020 Population by Census Block Groups

**Fig. 5.**
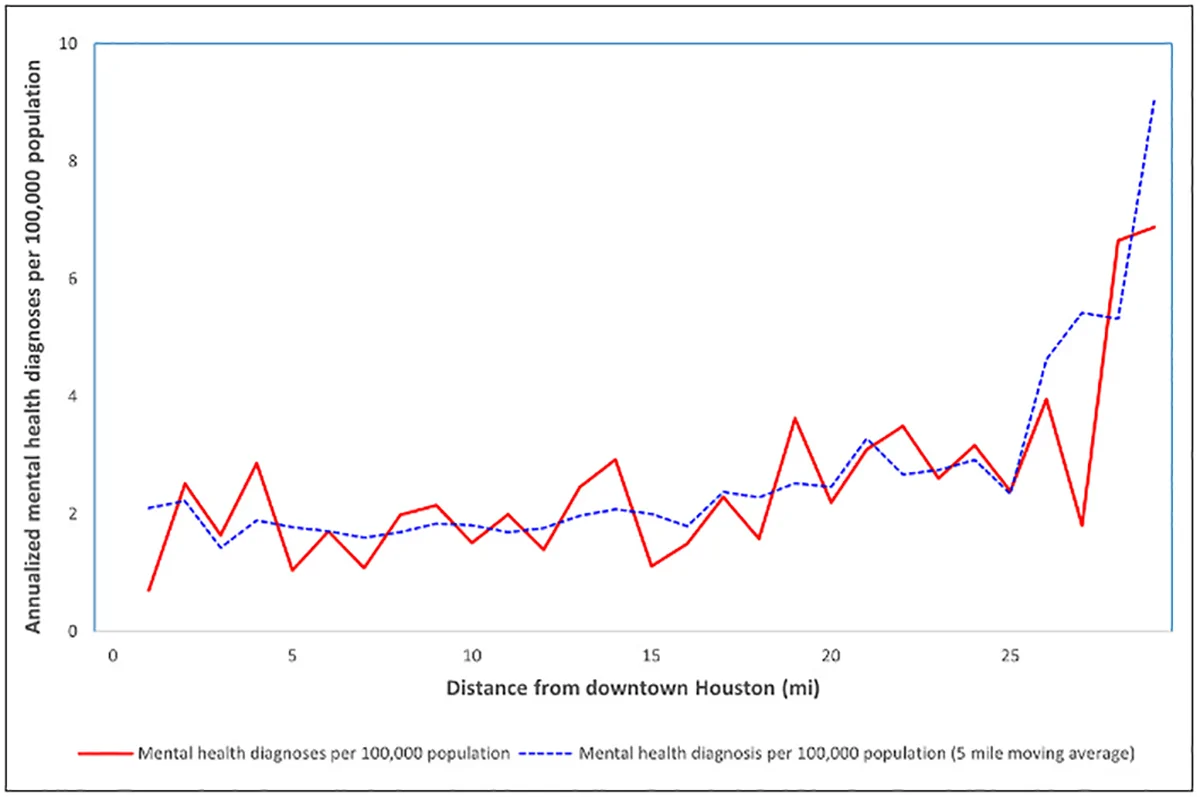
Mental Health Diagnosis (per 100,000 population) for Those Dying by Firearm Suicide by Distance from Downtown Houston

**Table 1:** Independent variables used in multivariate modeling.

Demographic characteristics	
AGE_65PLUS	Persons, ages 65 or older, 2020
WHITE	White non-Hispanic persons, 2020
BLACK	Black/African-American non-Hispanic persons, 2020
HISPANIC	Hispanic persons, 2020
Economic characteristics	
MED_HH_INC	Estimated median household income, 2020
ADI	Area Disadvantage Index, 2018
PCT_POV	Percent of population living in poverty, 2018
UNEMP_RATE	Unemployment rate, 2018
Educational characteristics	
EDU_HIGH_S	Persons with only high school education, 2018
Land use characteristics	
COMMERCIAL	Whether block group contains commercial area
INDUSTRIAL	Whether block group contains industrial area
CHURCH	Number of churches in block group, 2020
BARS	Number of bars in block group, 2020
FASTFOOD	Number of fast food businesses in block group, 2020
GAS_STATN	Number of gas stations in block group, 2020
GUN_STORE	Number of gun stores in block group, 2020
HOSPITAL	Whether block group contains a hospital
PARK_LOTS	Number of parking lots in block group, 2020
PARKS	Whether block group contains a park
VACANT	Number of vacant units in block group, 2020
Community cohesion	
PCT_VOTE16	Percent of voting age population who voted, 2016
Geographical separation	
D_DWNTOWN	Distance to downtown Houston (mi)

**Table 2:** Regression models of firearm suicides by census block groups.

Dependent variable:	Log of number of firearm suicides per block group	
	A	B
	Full	Reduced
	Model	Model
N:	2830 block groups	2830 block groups
Df:	2805	2824
Type of Model	Poisson-Gamma	Poisson-Gamma
No. of samples:	500,000	500,000
No. of ‘burn in’ samples:	400,000	400,000
Log likelihood:	−1717.48	−1741.51
AIC:	3482.97	3493.01
Mean Absolute Deviation:	0.37	0.38
Mean-squared Predictive Error:	0.25	0.26
Dispersion multiplier:	0.02	0.04

Modeled parameters	Full model		Reduced model	
	Coefficient	Tolerance	Coefficient	Tolerance
Exposure/offset variable				
POP 2020	1.0000	–	1.0000	–
INTERCEPT	−9.8099***	–	−9.2467***	–
Demographic characteristics				
AGE_65PLUS	0.0004	0.69	–	–
WHITE	−0.00004	0.33	–	–
BLACK	−0.0003*	0.75	–	–
HISPANIC	−0.0003***	0.56	–	–
Economic characteristics				
MED_HH_INC	−0.000002	0.46	–	–
ADI	−0.0003	0.59	–	–
PCT_POV	0.0262	0.98	0.0301*	1.00
UNEMP_RATE	0.0238	0.98	–	–
Educational characteristics				
EDU_HIGH_SCHOOL	0.0002	0.47	–	–
Land use characteristics				
COMMERCIAL	0.1835	0.97	–	–
INDUSTRIAL	0.3481	0.97	–	–
CHURCH	−0.0563	0.94	–	–
BARS	0.1057**	0.72	0.1110***	0.99
FASTFOOD	0.0085	0.75	–	–
GAS_STATN	0.0246	0.84	–	–
GUN_STORE	0.0001	0.99	–	–
HOSPITAL	0.0898	0.98	–	–
PARK_LOTS	−0.0304	0.72	–	–
PARKS	−0.0929	0.99	–	–
VACANT	0.0001	0.74	–	–
Community cohesion				
PCT_VOTE16	0.0147***	0.35	–	–
Geographical separation				
D_DWNTOWN	0.0187**	0.64	0.0214***	0.99

* *p* ≤ .05.

** *p* ≤ .01.

*** *p* ≤ .001.

<blank> Not significant.

## Data Availability

The authors do not have permission to share data.

## Appendix A. Poisson-gamma exposure model of firearm suicides

The regression model followed the logic first formulated by Besag et al. (1995). Formally, for each block group the number of firearm suicides per capita is assumed to be Poisson distributed and independent, and has the form:

(A1)
$$y_{i}|\lambda_{i}\sim \text{Poisson}\left(\lambda_{i}\right)$$

with the mean organized as:

(A2)
$$\mu_{i}=v_{i}\lambda_{i}$$

where *v*_*i*_ is the *exposure* measure and *λ*_*i*_ is the *rate* (or risk). The exposure variable is the baseline variable – population, to which the number of firearm suicides is related.

The rate is further structured as a Poisson-Gamma-Exposure model:

(A3)
$$\mu_{i}=v_{i}\lambda_{i}=v_{i}\cdot exp\left({\text{x}}_{i}^{T}\beta +\varepsilon_{i}\right)$$

where exp.() is an exponential function, **β** is a vector of unknown coefficients for the *k* covariates plus an intercept, and *ε*_i_ is the model error independent of all covariates. The error, exp(*ε*_i_), is assumed to follow a Gamma distribution with a mean equal to 1 and a variance equal to *τ*=1/*ψ* where *ψ* is a parameter that is greater than 0 (Lord, 2006; Cameron & Trivedi, 2013).

In practice, this means that the logarithm of population is treated as a fixed-effect independent variable since the logarithm of a ratio is equal to the difference in the logarithms:

(A4)
$$Ln\left(\frac{S_{i}}{P_{i}}\right)=Ln\left(S_{i}\right)-Ln\left(P_{i}\right)=\alpha +\sum_{j,i}\beta_{k}X_{i}+\varepsilon_{i}$$

where *S*_*i*_ is the number of firearm suicides for a block group, *P*_*i*_ is the population of the block group, *X*_*i*_ is one of *“i”* independent variables shown in Table 1 for a block group, *β*_*i*_ is the coefficient associated with the independent variable, and *ε*_*i*_ is the model error for a block group independent of all covariates.

The logarithm of population is then subtracted from both sides of the equation to place it on the right side:

(A5)
$$Ln\left(S_{i}\right)=Ln\left(P_{i}\right)+\alpha +\sum_{j,i}\beta_{k}X_{ki}+\varepsilon_{i}$$

Note that there is no coefficient for the logarithm of population (i.e., it has a coefficient of 1.0). Further, exponentiating each side, we have the final equation:

(A6)
$$S_{i}=P_{i}e^{\alpha +\sum_{j,i}\beta_{k}X_{i}+\varepsilon_{i}}$$

In other words, the model estimates the number of firearm suicides per capita given the independent variables shown in Table 1 but in an exposure form. It is a general model that handles both counts and rates for a Poisson-based model (Besag et al., 1995).

Because of the complexity of the model, it was estimated with a Markov Chain Monte Carlo (MCMC) simulation, using the CrimeStat software package (Levine, 2013). The number of simulation runs was 500,000 with the initial 400,000 runs being discarded (called ‘Burn in’). The Gelman-Rubin statistic was used to determine whether the ‘Burn in’ sequence had achieved equilibrium (Gelman & Rubin, 1992). The final output was based on the last 100,000 samples with the mean taken as the best estimate of the parameter.

## References

[R1] Akbarizadeh F, Hajivandi A, Hajivandi M, & Zeidabadinejad MS. (2019). Marriage related suicide fatality rates. Iranian Journal of Psychiatry, 14(1), 54–59. February https://www.researchgate.net/publication/330312503_Marriage_Related_Suicide_Fatality_Rates. PMC6505054 31114618

[R2] Alonso W. (1964). Location and land use: Towards a general theory of land rent. Cambridge, MA: Harvard University Press.

[R3] Ames DL & McClelland LF. (2002). Historic residential suburbs. National Register Bulletin, September. National Register of historic places, National Park Service, U.S. Department of the Interior: Washington, DC. https://www.nps.gov/subjects/nationalregister/upload/NRB46_Suburbs_part1_508.pdf.

[R4] Bertuccio, (2024). Global trends in youth suicide from 1990 to 2020: An analysis of data from the WHO mortality database. eClinicalMedicine, 70, Article 102506. https://www.thelancet.com/journals/eclinm/article/PIIS2589-5370(24)00085-3/fulltext. 10.1016/j.eclinm.2024.102506 PMC10911948 38440131

[R5] Besag J, Green P, Higdon D, & Mengersen K. (1995). Bayesian computation and stochastic systems (with discussion). Statistical Science, 10, 3–66.

[R6] Brådvik L. (2018). Suicide risk and mental disorders. International Journal of Environmental Research and Public Health, 15(9), 2028. https://www.ncbi.nlm.nih.gov/pmc/articles/PMC6165520. 10.3390/ijerph15092028 PMC6165520 30227658

[R7] Branas CC, Nance ML, Elliot MR, Richmond TS, & Schwab CW. (2004). Urban-rural shifts in intentional firearm death: Different causes, same results. American Journal of Public Health, 94(10), 1750–1755. 10.2105/ajph.94.10.1750, 2004 Oct https://pubmed.ncbi.nlm.nih.gov/15451745/. PMC1448529 15451745

[R8] Calati R, Ferrari C, Brittner M, Oasi O, Oliè E, Carvalho AF, Courtet P. Suicidal thoughts and behaviors and social isolation: A narrative review of the literature. Journal of Affective Disorders 2019 Feb 15;245:653–667. doi:10.1016/j.jad.2018.11.022. Epub 2018 Nov 7. https://pubmed.ncbi.nlm.nih.gov/30445391/. 30445391

[R9] Cameron AC, & Trivedi PK. (2013). Regression analysis of count data (Second Edition). Cambridge, U.K: Cambridge University Press.

[R10] Case A, & Deaton A. (2017). Mortality and morbidity in the 21st century. In Brookings Papers on Economic Activity. Spring. https://www.brookings.edu/articles/mortality-and-morbidity-in-the-21st-century/. 10.1353/eca.2017.0005 PMC5640267 29033460

[R11] Case A, & Deaton A. (2023). Without a college degree, life in America is staggeringly shorter. New York Times, Oct., 3, 2023. https://www.nytimes.com/2023/10/03/opinion/life-expectancy-college-degree.html.

[R12] CDC. (2022). Reducing military and veteran suicide, congressional testimony, June 15, 2022. Centers for Disease Control and Prevention: Atlanta, GA. https://www.cdc.gov/washington/testimony/2022/t20220615.htm.

[R13] CDC. (2023). Suicide data and Statistics. Centers for Disease Control and Prevention: Atlanta, GA. https://www.cdc.gov/suicide/facts/data.html.

[R14] CDC. (2024a). Facts about suicide. Centers for Disease Control and Prevention: Atlanta, GA. https://www.cdc.gov/suicide/facts/index.html.

[R15] CDC (2024b). Firearm suicide trends. Firearm injury and death prevention. Centers for Disease Control and Prevention: Atlanta, GA. https://www.cdc.gov/firearm-violence/php/data-trends/firearm-suicide-data-trends.html.

[R16] Cervero R. (1993). Surviving in the suburbs: Transit’s untapped frontier. Access, Spring (2). https://www.accessmagazine.org/spring-1993/transits-untapped-frontier/.

[R17] Couillard BK, & Foote CL. (2019). Recent employment growth in cities, suburbs, and rural communities. Working Paper No 19–20,. Boston, MA: Federal Reserve Bank of Boston https://www.bostonfed.org/publications/research-department-working-paper/2019/recent-employment-growth-in-cities-suburbs-and-rural-communities.aspx.

[R18] Cox W. (Aug 2018). Dispersed American form yields quick work trips. New Geography, 8. https://www.newgeography.com/content/006051-the-dispersed-city.

[R19] Cuesta I, Montesó-Curto P, Metzler Sawin E, (2021). Risk factors for teen suicide and bullying: An international integrative review. International Journal of Nursing Practice, 27(3), Article e12930. 10.1111/ijn.12930. https://pubmed.ncbi.nlm.nih.gov/33830586/33830586

[R20] Curtain SC & Garnett MF. (2023). Suicide and homicide death among youth and young adults aged 10–24: United States, 2001–2021. NCHS Data Brief, 471, June. https://www.cdc.gov/nchs/products/databriefs/db471.htm. 37367034

[R21] DeAngelis T. (2022). Veterans are at a higher risk for suicide. Psychologists are helping them tackle their unique struggles. Monitor on psychology, November. American Psychological Association: Washington, DC. https://www.apa.org/monitor/2022/11/preventing-veteran-suicide.

[R22] Durkheim E. (1897). Suicide: A study in sociology. Translated by Spaulding JA & Simpson George. 1951. The Free Press: New York.

[R23] Egan DJ, & Nield K. (2000). Towards a theory of intraurban hotel location. Urban Studies, 37(3), 611–621. https://econpapers.repec.org/article/saeurbstu/v_3a37_3ay_3a2000_3ai_3a3_3ap_3a611-621.htm.

[R24] Fakhari A, Azizi H, Farahbakhsh M, & Esmaeli ED. (2022). Effective programs on suicide prevention: Combination of review of systematic reviews with expert opinions. International Journal of Preventive Medicine, March 12. 13: 39. https://pmc.ncbi.nlm.nih.gov/articles/PMC9069150/. 10.4103/ijpvm.IJPVM_454_20 PMC9069150 35529514

[R25] Filatova T, Parker D, & van der Veen A. (2009). Agent-based urban land markets: Agent’s pricing behavior, land prices, and urban land use change. Journal of Artificial Societies and Social Simulation, 12(13). https://jasss.soc.surrey.ac.uk/12/1/3.html.

[R26] Fry R. (2020). Prior to COVID-19, urban core counties in the U.S. were gaining vitality on key measures. In Report, July 29. Washington, DC: Pew research center. https://www.pewresearch.org/social-trends/2020/07/29/prior-to-covid-19-urban-core-counties-in-the-u-s-were-gaining-vitality-on-key-measures/.

[R27] Gallup (2020). What percentage of Americans own guns? The Short Answer, Gallup, Inc.: Washington, DC. https://news.gallup.com/poll/264932/percentage-americans-own-guns.aspx.

[R28] Gelman A, & Rubin DB. (1992). Inference from iterative simulation using multiple sequences (with discussion). Statistical Science, 7, 457–511.

[R29] Ghadapasha M, Talaie R, Mahmoodi Z, Karimi SE, Forouzesh M, Morsalpour M, … Dadashzadehasl N. (2024). Spatial, geographic and demographic factors associated with adolescent and youth suicide: A systematic review study. Frontiers in Psychiatry, 15, Article 1261621. https://www.ncbi.nlm.nih.gov/pmc/articles/PMC10893588/. 10.3389/fpsyt.2024.1261621 PMC10893588 38404471

[R30] Gilderbloom JI, Riggs WW, & Meares WL. (2015). Does walkability matter? An examination of walkability’s impact on housing values, foreclosures, and crime. Cities, 42 (Part A), February, 13–24.

[R31] Gloger S, Martinez P, Behn A, Chacon MV, Cottin M de Medina DD, & Vőhringer PA. (2021). Population-attributable risk of adverse childhood experiences for high suicide risk, psychiatric admissions, and recurrent depression, in depressed outpatients. European Journal of Psychotraumatology, 12(1), 1874600. https://www.ncbi.nlm.nih.gov/pmc/articles/PMC8118528. 10.1080/20008198.2021.1874600 PMC8118528 34025917

[R32] Goldberger AS. (1991). Chapter 23: Multicollinearity. In Goldberger AS. (Ed.), A course in econometrics. Cambridge, MA: Harvard University Press.

[R33] Greater Houston Partnership (2024). Explore the region. Fact sheet. Greater Houston Partnership: Houston, TX. https://www.houston.org/why-houston/explore-regions?county=harris.

[R34] Harne O. (2018). Geography of suicide: A look at the CDC’s recent report. Geolounge, (June 23). https://www.geolounge.com/geography-of-suicide-a-look-at-the-cdcs-recent-report/.

[R35] Heu U, Bogren M, Wang AG, & Brådvik L. (2018). Aspects of additional psychiatric disorders in severe depression/melancholia: A comparison between suicides and controls and general pattern. International Journal of Environmental Research and Public Health, 15(7), 1299. Jun 21 https://www.ncbi.nlm.nih.gov/pmc/articles/PMC6068998. 10.3390/ijerph15071299 PMC6068998 29933593

[R36] H-GAC. (2024). The area agency on aging. Houston-Galveston Area Council: Houston, TX. https://www.h-gac.com/area-agency-on-aging.

[R37] Hopkins J. (2023). CDC provisional gun data: Gun suicides reach all-time high in 2022, gun homicides down slightly from 2021. Baltimore, MD: News Item, Johns Hopkins Bloomberg School of Public Health, Johns Hopkins. https://publichealth.jhu.edu/center-for-gun-violence-solutions/2023/cdc-provisional-data-gun-suicides-reach-all-time-high-in-2022-gun-homicides-down-slightly-from-2021.

[R38] Houston Chronicle. (2024). Houston metro population grows by 140,000, but not enough to beat Dallas. March 15 https://www.houstonchronicle.com/business/article/houston-metro-growth-among-largest-nationally-19015814.php.

[R39] Houston State of Health (2023). 2023 demographics. Houston state of health data portal. Houston health department, Harris County public health department, Harris health system, Houston Methodist hospital system, and memorial Herman Hospital system: Houston, TX. https://www.houstonstateofhealth.com/demographicdata?id=2675&sectionId=935.

[R40] Hudson CG, & Doogan NJ. (2019). The impact of geographic isolation on mental disability in the United States. SSM – Population Health, August, 8, 100437. 10.1016/j.ssmph.2019.100437 PMC6626110 31338410

[R41] Ingraham C. (2018). Mapping the tide of suicide deaths across the United States. Washington post, may 24. https://www.washingtonpost.com/news/wonk/wp/2018/05/24/mapping-the-rising-tide-of-suicide-deaths-across-the-united-states/?utm_term=.0c32c60efa2b.

[R42] Inskip HM, Harris EC, & Barraclough B. (1998). Lifetime risk of suicide for affective disorder, alcoholism, and schizophrenia. The British Journal of Psychiatry, 172, 35–37. https://pubmed.ncbi.nlm.nih.gov/9534829/. 10.1192/bjp.172.1.35 9534829

[R43] Isaacs A. (2024). Making transit work in the suburbs. Streets, (January 23). https://streets.mn/2024/01/23/transit-in-the-suburbs/.

[R44] JAMA (2017). US suicide rates display growing geographic disparity. Journal of the American Medical Association, 2017; 317 (16):1616. https://www.ncbi.nlm.nih.gov/pubmed/28444288. 10.1001/jama.2017.4076 28444288

[R45] Kanji GK. (2006). 100 statistical tests (3rd ed., p. 26). Thousand Oaks, CA: Sage Publications.

[R46] Kegler SR, Stone DM, & Holland KM. (2017). Trends in suicide by level of urbanization - United States, 1999–2015. Morbidity and Mortality Weekly Report, 66 (10), March 17. 270–273. https://www.cdc.gov/mmwr/volumes/66/wr/mm6610a2.htm. 10.15585/mmwr.mm6610a2 PMC5657870 28301448

[R47] Kivisto AJ. (2022). Beyond legislative lethal means restriction approaches to suicide prevention. J. American Academy Psychiatry & the Law, 50 (2), 170–176. https://jaapl.org/content/50/2/170. 10.29158/JAAPL.210140-21 35668037

[R48] Kneebone E & Berube A. (2023). Post-pandemic poverty is rising in America’s suburbs. Research report: October 11, 2023, Brookings Institution: Washington, DC. https://www.brookings.edu/articles/post-pandemic-poverty-is-rising-in-americas-suburbs.

[R49] Kneebone E, & Raphael S. (2011). City and suburban crime trends in metropolitan America. Research report,. Brookings Institution: Washington, DC: Metropolitan Opportunity Series, May https://www.brookings.edu/wp-content/uploads/2016/06/0526_metropolitan_crime_kneebone_raphael.pdf.

[R50] Lehman CF. (2020). The role of marriage in the suicide crisis. Blog, Institute for Family Studies: Charlottesville, VA. https://ifstudies.org/blog/the-role-of-marriage-in-the-suicide-crisis.

[R51] Leigh-Hunt N, Bagguley D, Bash K, Turner V, Turnbull S, Valtorta N, (2017). An overview of systematic reviews on the public health consequences of social isolation and loneliness. In Public health, 152, 157–171. Washington, DC: National Library of Medicine. https://pubmed.ncbi.nlm.nih.gov/28915435/. 10.1016/j.puhe.2017.07.035 28915435

[R52] Levine N. (2011). Spatial variation in motor vehicle crashes by gender in the Houston metropolitan area. Proceedings of the 4^th^ international conference on Women’s issues in transportation. Volume II: Technical papers, Transportation Research Board: Washington, DC. 12–25. http://onlinepubs.trb.org/onlinepubs/conf/cp46v2.pdf.

[R53] Levine N. (2013). CrimeStat: A spatial statistics program for the analysis of crime incident locations (version 4.02). July. Washington, DC: National Institute of Justice. http://www.nij.gov/CrimeSta.

[R54] Levine N, Drake SA, Reynolds TF, Yang Y, Wolf DA, Persse D, … Holcomb JB. (2020). Spatial correlates of firearm-related deaths in Harris County. TX. Homicide Studies., 25(1), 37–60. https://journals.sagepub.com/eprint/ZJQNHXSXRZJDZPFZSYKE/full#.

[R55] Lord D. (2006). Modeling motor vehicle crashes using Poisson-gamma models: Examining the effects of low sample mean values and small sample size on the estimation of the fixed dispersion parameter. Accident Analysis & Prevention, 38(4), 751–766. https://www.sciencedirect.com/science/article/abs/pii/S0001457506000169. 10.1016/j.aap.2006.02.001 16545328

[R56] Lynch L, Mielenz TJ, Li G, Eby DW, Molnar LJ, Betz ME, DiGuiseppi C, Hill LL, Jones V. & Strogatz D. (2021). Rate of social isolation by geographic location among older adults. Brief research report on aging and public health, Frontiers in Public Health, December. 9: 791683. National Library of medicine: Washington, DC. https://www.ncbi.nlm.nih.gov/pmc/articles/PMC8702723/. 10.3389/fpubh.2021.791683 PMC8702723 34957037

[R57] Mann JJ, & Michel CA. (2016 Oct 1). (2016). Prevention of firearm suicide in the United States: What works and what is possible. The American Journal of Psychiatry, 173(10), 969–979. 10.1176/appi.ajp.2016.16010069 27444796

[R58] Mascia J & Brownlee C. (2023). How many guns are circulating in the U.S.? The trace, March 6. https://www.thetrace.org/2023/03/guns-america-data-atf-total/.

[R59] McLoughlin RJ, Hazeltine M, Dacier BM, Hirsh MP, Cleary MA, & Aidlen JT. (2019). (2019). Young lives cut short: The relationship between firearms and pediatric suicide. Journal of Trauma and Acute Care Surgery, 87(1), 161–167. https://pubmed.ncbi.nlm.nih.gov/30882762/. 10.1097/TA.0000000000002257 30882762

[R60] Merrick MT, Ford DC, Ports KA, Guinn AS, Chen J, Klevens J, … Mercy JA. (2019). Vital signs: Estimated proportion of adult health problems attributable to adverse childhood experiences and implications for prevention — 25 states, 2015–2017. MMWR. Morbidity and Mortality Weekly Report, 68, 999–1005. https://www.cdc.gov/mmwr/volumes/68/wr/mm6844e1.htm?s_cid=mm6844e1_w. 10.15585/mmwr.mm6844e1 PMC6837472 31697656

[R61] Miller M, Azrael D, & Barber C. (2012). Suicide mortality in the United States: The importance of attending to method in understanding population-level disparities in the burden of suicide. Annual Review of Public Health, 33, 393–408, 2012 Apr https://pubmed.ncbi.nlm.nih.gov/22224886/. 10.1146/annurev-publhealth-031811-124636 22224886

[R62] Motillon-Toudic C, Walter M, Sèguin M, Carrier JD, Berrouiguet S, Lemey C. Social isolation and suicide risk: Literature review and perspectives. European Psychiatry 2022 Oct 11;65(1):e65. doi: 10.1192/j.eurpsy.2022.2320. https://www.ncbi.nlm.nih.gov/pmc/articles/PMC9641655/. PMC9641655 36216777

[R63] Naik-Mathuria B, Cain CM, Elizabeth A, Chen L, & Pompeii LA. (2023). Defining the full Spectrum of pediatric firearm injury and death in the United States: It is even worse than we think. Annals of Surgery, 278(1), 10–16, July. https://pubmed.ncbi.nlm.nih.gov/36825500/. 10.1097/SLA.0000000000005833 PMC10249597 36825500

[R64] Nicolaides B & Wiese A. (2017). Suburbanization in the United States after 1945. American history, Oxford research encyclopedia. April 26. 10.1093/acrefore/9780199329175.001.0001/acrefore-9780199329175-e-64.

[R65] NIJ. (2014). Practice profile: Reducing gun violence. In Crime solutions. Justice. Washington, DC: National Institute of Justice, Office of Justice Programs, U.S. Department of. https://crimesolutions.ojp.gov/ratedpractices/33.

[R66] Pate A. (2014). Social isolation: Its impact on the mental health and wellbeing of older Victorians (Australia). https://cotavic.org.au/wp-content/uploads/2019/05/Working-Paper_Social-Isolation.pdf.

[R67] Pruitt LD, Smolenski DJ, Bush NE, Tucker J, Issa F, & Hoyt TV. (2019). Suicide in the military: Understanding rates and risk factors across the United States’ armed forces. Military Medicine, 184(1 Suppl), 432–437. https://pubmed.ncbi.nlm.nih.gov/30423136/. 10.1093/milmed/usy296 30423136

[R68] REDCAP. (2023). Research electronic data capture. Nashville, TN: Vanderbilt University. https://projectredcap.org.

[R69] Reiman E. (2023). Poverty is rising faster in American suburbs as lower-income people are pushed out of big cities. Business Insider, October, 19, 2023. https://www.businessinsider.com/suburban-areas-poverty-rising-faster-than-cities-post-pandemic-2023-10.

[R70] Rockefeller Foundation (2013). Suburban poverty in the United States. Research report. May. https://www.rockefellerfoundation.org/wp-content/uploads/Suburban-Poverty-in-the-United-States.pdf.

[R71] Rossen LM, Hedegaard H, Khan D, & Warner M. (2018). County-level trends in suicide rates in the U.S., 2005–2015. American Journal of Preventive Medicine, 55 (1), July, 72–79. https://www.sciencedirect.com/science/article/pii/S0749379718316775. 10.1016/j.amepre.2018.03.020 PMC6038117 29773489

[R72] Santini ZI, Koyanagi A, Tyrovolas S, Mason C, & Haro JM. (2015). The association between social relationships and depression: A systematic review. In Journal of affective disorders, 175(2015), 53e65. Washington, DC: National Library of Medicine. https://pubmed.ncbi.nlm.nih.gov/25594512/. 10.1016/j.jad.2014.12.049 25594512

[R73] Schwarzbach M, Luppa M, Forstmeier S, König H, & Riedel-Heller SG. (2014). Social relations and depression in late life-a systematic review. International journal of geriatric psychiatry, 29, 1–21. National Library of Medicine: Washington, DC. https://pubmed.ncbi.nlm.nih.gov/23720299/. 10.1002/gps.3971 23720299

[R74] Shearer C, Vey JS, & Kim J. (2019). Where jobs are concentrating and why it matters to cities and regions. In Brookings, June 18. Washington, DC: Brookings Institution. https://www.brookings.edu/articles/where-jobs-are-concentrating-why-it-matters-to-cities-and-regions/#group-seqs-3.

[R75] Smart R, Morral AR, Ramchand R, Charbonneau A, Williams J, Smucker S, … Xenakis L. (2024). The science of gun policy: A critical synthesis of research evidence on the effects of gun policies in the United States. Santa Monica: Gun Policy Initiative, RAND Corporation. https://www.rand.org/pubs/research_reports/RRA243-9.html.

[R76] Solomon A. (2024). Why aren’t modern suburbs built on a walkable grid? How stuff works?. https://science.howstuffworks.com/engineering/civil/why-so-many-suburban-streets-twist-and-turn.htm.

[R77] SPRC. (2019). Suicide by age. Washington, DC: Suicide Prevention Resource Center. http://www.sprc.org/scope/age.

[R78] Stanley IH, Hom MA, Rogers ML, Antestis MD, & Joiner TE. (2017). Discussing firearm ownership and access as part of suicide risk assessment and prevention: “Means safety” versus “means restriction”. Archives of Suicide Research, Apr-Jun, 21(2), 237–253. https://pubmed.ncbi.nlm.nih.gov/27077214/. 10.1080/13811118.2016.1175395 27077214

[R79] Stevens E, Carson R, & Wall L. (2024). Factors, dynamics, and effects of isolation for older people: An exploratory study. Research report, Australian Institute of Family Studies: Southbank, Australia. https://aifs.gov.au/sites/default/files/2024-02/2311_FL_Isolation-older-people-final-report.pdf.

[R80] Studdert DM, Zhang Y, Swanson SA, Prince L, Rodden JA, Holsinger EE, … Miller M. (2020). Handgun ownership and suicide in California. New Eng J Med, 382, 2220–2229. 10.1056/nejmsa1916744 32492303

[R81] Tomer A, & George C. (2023). Building for proximity: The role of activity centers in reducing total miles traveled. In Research, June 29. Washington, DC: Brookings Institution. https://www.brookings.edu/articles/building-for-proximity-the-role-of-activity-centers-in-reducing-total-miles-traveled/.

[R82] Tulane. (2020). Understanding the effects of social isolation on mental health. Report, December 8. New Orleans: School of Public Health and Tropical Medicine, Tulane University. https://publichealth.tulane.edu/blog/effects-of-social-isolation-on-mental-health/.

[R83] U.S. Census Bureau (2022). Redefining urban areas following the 2020 Census. U.S. Census Bureau, U.S. Department of Commerce: Washington, DC. https://www.census.gov/newsroom/blogs/random-samplings/2022/12/redefining-urban-areas-following-2020-census.html.

[R84] U.S. Census Bureau (2023). Quick Facts: Harris County, Texas. U.S. Census Bureau, U.S. Department of Commerce: Washington, DC. https://www.census.gov/quickfacts/harriscountytexas.

[R85] VA. (2022). National veteran suicide prevention annual report. Washington, DC: Office of Mental Health and Suicide Prevention, Department of Veterans Affairs. https://www.mentalhealth.va.gov/docs/data-sheets/2022/2022-National-Veteran-Suicide-Prevention-Annual-Report-FINAL-508.pdf.

[R86] Veazie S, Gilbert J Winchell K, Paynter R, & Guise J-M. (2019). Addressing social isolation to improve the health of older adults: A rapid review. Agency for Health Care Research and Quality, no. 19-EHC009-EF. Scientific resource center: Portland, OR. National Library of medicine: Washington, DC. https://pubmed.ncbi.nlm.nih.gov/30830728/. 30830728

[R87] VTO. (2012). Fact #759: December 12, 2024 rural vs. urban driving differences, vehicle technologies office. Office of Energy Efficiency & Renewable Energy. Washington, DC: U. S. Department of Energy. https://www.energy.gov/eere/vehicles/fact-759-december-24-2012-rural-vs-urban-driving-differences.

[R88] Webster DM, & Wintemute GJ. (2015). Effects of policies designed to keep firearms from high-risk individuals. Annual Review of Public Health, 36, 21–37. 10.1146/annurev-publhealth-031914-122516, 2015 Mar 18. Epub 2015 Jan 7 https://pubmed.ncbi.nlm.nih.gov/25581152/. 25581152

[R89] WHO. (2022). Mental disorders. Fact Sheets, World Health Organization: Geneva, Switzerland. https://www.who.int/news-room/fact-sheets/detail/mental-disorders.

[R90] Xing Y, Rodier C, Harold B, & Sintetos M. (2022). New mobility for sustainable suburban and rural travel. In News item, environmental justice efforts, Institute of Transportation Studies, University of California. Davis: Davis, CA. https://its.ucdavis.edu/blog-post/new-mobility-for-sustainable-suburban-and-rural-travel/.

[R91] Zuvanich A. (2023). Houston-area suburbs growing much faster than city since 2020. News item, Houston Public Media, (December 8). https://www.houstonpublicmedia.org/articles/news/local/2023/12/08/471915/houston-area-suburbs-growing-much-faster-than-city-in-wake-of-pandemic/.

